# Risk Analysis and Extension Assessment for the Stability of Surrounding Rock in Deep Coal Roadway

**DOI:** 10.3390/ijerph16234752

**Published:** 2019-11-27

**Authors:** Chunjing Gao, Dongmei Huang, Xikun Chang, Han Xi

**Affiliations:** 1 State Key Laboratory of Mining Disaster Prevention and Control Co-Founded by Shandong Province and the Ministry of Science and Technology, Shandong University of Science and Technology, Qingdao 266590, China; gthdyx0316@163.com (C.G.); xh_12_12@163.com (H.X.); 2 College of Mining and Safety Engineering, Shandong University of Science and Technology, Qingdao 266590, China; 3 National Demonstration Center for Experimental Mining Engineering Education, Shandong University of Science and Technology, Qingdao 266590, China

**Keywords:** stability of surrounding rock, deep roadway, risk assessment, extension method

## Abstract

In order to evaluate the surrounding rock stability of deep roadways, the diversity of accident hazard sources in deep coal mining is statistically analyzed. To conduct an effective evaluation, first, the risk analysis of the factors affecting the rock mass accidents is carried out, and the comprehensive safety index system of rock accidents in deep mine roadway is established. Further, combining the theory of hazard sources with the extension method, a matter–element model for the risk assessment of rock mass accidents in deep roadway is established. Finally, the hazard sources for the surrounding rock stability of deep roadway in the E-Zhuang coal mine of Xinwen Ming area are evaluated. The results show that the risk grade of the surrounding rock for deep roadways in E-Zhuang coal mine is “B”, which is generally safe, the human factors and organizational management factors are relatively safe, and some suggestions for improvement are put forward.

## 1. Introduction

With the development of mine production in China, the depth of coal mines gradually increased, and the ecological environment developed complex conditions. Underground mining with a depth of more than 600 m is called deep mining. In China, deep mining is increasingly being adopted. Deep mining is different from shallow mining, for example: more ground pressure, more gas, and faster deformation rate [1]. These factors affect the safety of coal mine production. Therefore, as the mining depth and breadth increase, the difficulty of mine rock engineering is also increasing. The deepening of mining depth and the improvement of the mechanization level have brought increasing difficulties regarding the stability of surrounding rock. In addition, the mining influence leads to exposure of weak rock surfaces to stress in the process of excavation and artificial disturbance, and then extends micro and macro cracks, until the whole rock mass is destroyed. The destroyed rock mass would cause the instability of surrounding rock and supporting problem, and bring about a series of environmental disasters, such as rock burst, mine roadway roof caving, impact ground pressure, or surface subsidence. These environmental disasters will seriously affect the safe production of coal enterprises. In China, from the year 2013 to 2017, about 70% of coal mine deaths were caused by the roof, gas, and machine electricity accidents, among them, roof accidents accounted for 39% of all coal mine accidents [2]. In 2018, nearly 35% of the coal mine safety accidents in China were caused by roof accidents [3]. To solve these problems, a comprehensive extension method should be proposed.

To reduce these environmental disasters in coal mines, the safety assessment of the surrounding rock is important in the process of mine design and construction. Meanwhile, reliable excavation and supporting could be provided by scientific and reasonable evaluation of the surrounding rock. As the premise of support, the stability evaluation of deep surrounding rock is an urgent problem to be solved. Therefore, it is very important to study the stability of surrounding rock in the whole mine, especially in the key production position.

Many scholars have done a lot of research in evaluating mine safety. For example, Andonov [4] evaluated the geo-mechanical conditions of the free passage construction of irrigation systems, and suggestions for the improvement of rock mass stability around mine workings were developed and used. Ural et al. [5] compared Turkey’s safety performance indicators with those of the mining industry in other major mining countries, enabling the authorities to develop strategies to take relevant precautions and improve the mining industry in Turkey. Li et al. [6] identified the danger sources before a coal mine accident and then gave pre-control measures and information monitoring methods based on the classification of hidden danger sources. Finally, risk warning and risk control methods were determined, and management standards and measures were used to eliminate hidden dangers. Based on the clustering analysis method and the fuzzy equivalence relation, Zhang et al. [7] studied the classification scheme of surrounding rock stability of the coal roadway in the Huozhou mining area and proposed the corresponding supporting countermeasures. Jiang et al. [8] comprehensively evaluated the stability of the surrounding rock of the No. 4 coal seam in Hujiahe Mine based on the rock quality designation (RQD) value, strength index, volume modulus, trimming modulus, and other methods. Huang et al. [9] used the analytic hierarchy process and gray correlation analysis to evaluate the risk of the deep surrounding rock in the E-Zhuang coal mine of the Chinese Xinwen Mining Area. Christopher et al. [10] summarized the experience of coal-fired accidents in the United States and internationally and described the risk factors in detail. A framework has been established that can be used to guide the risk assessment process. Huang et al. [11] established a gray-level assessment (G-A) model for gas explosion accidents and combined it with specific coal mine examples to determine the risk level and key prevention issues. Liu et al. [12] established a gas explosion accident tree and, based on the FTA-AHP analysis results, combined it with China’s coal industry safety production related policies and regulations to conduct safety assessment of coal mine gas accidents. Peng et al. [13] developed an innovative multi-standard decision method (MCDM) to address coal mine safety assessment issues. Javadi et al. [14] developed a numerical model for analyzing the risk of coal explosion to map the high-risk areas of the Iranian Tabass mine. Glont et al. [15] established an information system to assess the security situation in the areas bordering the mining target; the system included information on the constructive and developmental characteristics of the mining target, and information generated by monitoring activities. Sun et al. [16] evaluated the rock properties using two heterogeneous carbonate rock samples and their corresponding multi-scale digital rock images and proposed a DRA up-scaling method.

Scholars have done a lot of research on deep roadway evaluation, which is widely used in methods and applications, but extension theory is rarely used in this area. The three elements of extension method are basic element theory, extension set theory, and extension logic. It can establish a matter–element model and seek the solution through various transformations quantitatively. Stability evaluation of the surrounding rock is the main basis of engineering design and calculation of support structures. Generally, a combination of qualitative and quantitative methods is used. Therefore, the application of extension to surrounding rock evaluation is a highly feasible research method, which can effectively solve engineering problems, increase the safety of mining engineering, and reduce accidents.

The rest of this article is organized as follows. First of all, we must consider all of the affecting factors simultaneously to conduct the index system of the risk assessment for surrounding rock, because rock stability is affected by many factors. Second, a comprehensive extension evaluation model of rock assessment is established in Section 3. Then an application example in the E-Zhuang coal mine of China is provided to illustrate the method; finally, the conclusions are drawn.

## 2. Evaluation Index System for Stability of Surrounding Rock of Deep Roadway

### 2.1. Identification of Risk Factors for Stability of Surrounding Rock in Deep Roadways

There are many factors that affect the stability of surrounding rock, such as technical equipment, operating environment, and operators [17]. In recent years, the fatal accidents in coal mine surrounding rock are basically the result of many factors, so these influencing factors need to be analyzed comprehensively. Many scholars have studied the factors. Zhang et al. [18] calculated the stable level of the dense seam roadway, he pointed out that the optimization control of surrounding rock needed to be improved and proposed some suggestions such as equipment and safety management. Wang [19] proposed a new type of support structure system that can effectively control the deformation of the surrounding rock. From the aspect of support equipment, the integrity, reliability, and safety of the equipment are the factors that must be considered in the evaluation of surrounding rock. Wen et al. [20] established the stress distribution model of roadway surrounding rock and analyzed the influence of mining depth and other environmental factors. Thus, environment is also an important factor to consider. According to the statistics, unsafe behavior leads to up to 95% of the serious and major accidents in coal mines [21]. In the stability of surrounding rock, safety management is an important problem that needs to be solved urgently. Some coal mine safety is poor, the level of safety management is low, the rules and regulations are not well implemented, these are the factors that cause accidents. Lu et al. [22] proposed reasonable measures and obtained the experience of the safety management of weak surrounding rock. Thus, the environment and equipment factors, the human factors, and the organizational management factors are all the main influential factors.

To sum up, from the perspective of safety evaluation and management, the stability analysis of the deep roadway should take into account the three aspects of human, environment and equipment, and organizational managements. Based on the actual situation of deep coal mine and the principle of evaluation index system, the three major hazard sources of this evaluation are determined as discussed below.

#### 2.1.1. Environment and Equipment Factors

In the deep roadway of a coal mine, the operating environment is rather complex, unsafe machinery and equipment are also the important reasons for roof accidents. Therefore, in this article, the environment and equipment factors refer to compression strength of the surrounding rock, the rock quality designation (RQD), the support equipment, the roof conditions, the hydrogeological conditions, the roadway’s size and shape, the depth of excavation, and the exploitation influence of workings nearby.

#### 2.1.2. Human Factors

Human factors refers to accidents caused by operators’ violation of basic operating procedures or inadequate supervision by supervisors. It is a significant reason of roof accidents in coal mines. People are the most uncontrollable factor in mining accidents. This study analyzed the safety awareness of the operator, the length of service, the artificial protection, the emergency self-rescue ability, and the staff certification.

#### 2.1.3. Organizational Management Factors

The management of a coal mine is very important. The wrong organizational management may bring out unpredictable losses to the construction. The organizational errors that may occur in coal mine safety management are summarized as rules and regulations, safety education and training, security management organization, emergency rescue plan, and safety technical measures.

**Figure 1:**
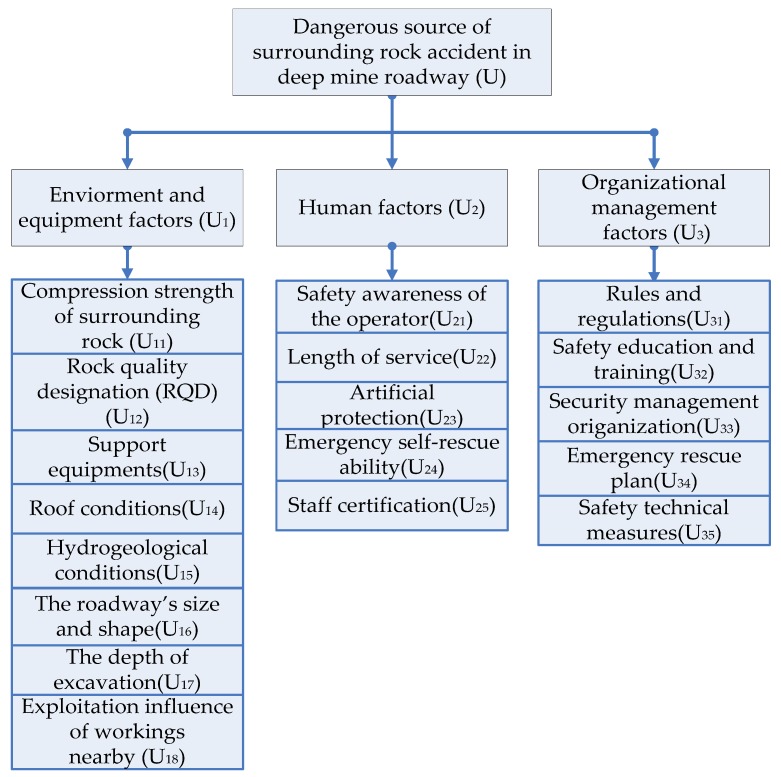
System of surrounding rock stability for deep roadway in coal mine [23].

According to the above risk identification results, an evaluation index system for surrounding rock stability of deep roadway was established, as shown in Figure 1.

### 2.2. Indices and the Related Explanations

The compressive strength of the surrounding rock is the limit of the external force that the surrounding rock can bear, it is the key factor to evaluate the stability of roadway surrounding rock engineering.

RQD is a quantitative index to measure rock quality, it can reflect the integrity of the rock mass by the rock quality. Therefore, it is an important influence factor in rock mass evaluation.

Mine support equipment mainly refers to the support equipment designed and manufactured to maintain the safety of the working-face roof in the process of underground mining. Its function is to provide a safe working environment for underground mining.

Roof refers to the rock layer located at a certain distance above the coal seam, roof conditions directly affect the safety of mine production.

Hydrogeological conditions mainly include coal seam structure, coal rock composition, rock fracture, fault, and groundwater. Once groundwater enters the work area through some channels, it will affect the safety of the mine and even cause hazards.

The roadway’s size and shape are the basis of roadway excavation construction and support. Generally, the roadway will be designed as a rectangle, trapezoid, and so on, because these are simple in structure and easy to construct. 

The depth of excavation is related to the stability of mining the geological layer, it is also an important factor that influences the rock stability.

Adjacent roadway mining will affect roadway support, resulting in roadway deformation, even leading to major accidents.

The safety consciousness level of the operator is an important external condition of the mine safety production. A higher level of safety awareness helps workers to solve emergencies more quickly and fully.

Length of service is an important aspect that affects the operator’s judgment and agility in solving problems, it is also the basis of technology, experience, and knowledge.

Artificial protection is a necessary measure in the process of coal mining, the quality of protective equipment is directly related to the work safety of workers.

Self rescue is the measures and methods taken by the workers in the disaster area to avoid disasters and protect themselves when accidents happen in the roadway. Self-rescue ability is a necessary skill for underground coal mining.

It is a most basic requirement that workers must be certified. Only after undergoing safety training and reaching the employment standard, all kinds of workers can work, otherwise, the professionalism and rationalization of the worker cannot be guaranteed.

Rules and regulations refer to the safety regulations and behavior constraints formulated by the mining area. They are the rules that all employees and managers must follow.

Safety education is the training and education of safety knowledge and skills for employees. It is also an important factor that influences the rock stability.

Security management organization is a kind of management organization of coal mine, its purpose is to ensure the smooth progress of mine production safety, implement a responsible system of production safety, and standardize the behavior of employees.

Emergency rescue plan refers to the action plan prepared in advance for possible accidents and quick emergency actions. It is an essential part of coal mine work.

Safety technical measures refer to the measures to eliminate the unsafe factors in mining, and realize the intrinsic safety of production, such as production process and mechanical equipment.

## 3. Stability Evaluation Model of Surrounding Rock in Deep Mine Roadway

### 3.1. Extension Evaluation Principle

Extenics is an original transversal subject [24,25,26] proposed by Cai Wen in 1983. It discusses the possibility of expanding problems, the rules and methods of developing and innovating, and is used to solve practical problems [27,28]. Its core is basic element theory, extension set, and extension logic. 

Multi-index extension comprehensive evaluation is a new method of multivariate data quantification based on matter–element model, extension set, and correlation function theory of extension science. It is a new method of multivariate data quantification decision-making created by extension scientists in China [29]. It is suitable for large-scale and multi-index safety quality evaluation and has a very broad prospect.

In this evaluation of surrounding rock stability, first, the safety evaluation index system is constructed, the weight value of each index is calculated by the method of gray correlation analysis, then, the correlation function is established, and the correlation of each index is calculated by the extension method. The correlation degree of each evaluation unit could be obtained from the weight and the correlation degree of the comprehensive index, and the safety level of the evaluation object can be determined according to the evaluation result; thus, the quantitative evaluation result can be obtained, and the corresponding solutions are proposed.

### 3.2. Establishment of Evaluation Model

#### 3.2.1. Determination of the Classic Domain

First of all, we must determine the classical domain to understand the matter. The matter is the primitive that represents the thing. The expression is M=(N,c,v), N is used to represent things, c indicates the attributes, conditions, functions, etc., v represents the corresponding value of this feature. The safety state of evaluation objects can be divided into j levels. The object to be evaluated can be expressed as:

(1) R=(N,c,v)

According to certain evaluation criteria, the security status of the evaluation object is divided into several levels, then the classical domain matter–element can be expressed as:

(2) $$R_{j}=(N_{j},C_{i},V_{j})=\left[\begin{array}{ccc} N_{j} & c_{1} & x_{j1}\\ & c_{2} & x_{j2}\\ & \cdots & \cdots \\ & c_{n} & x_{jn} \end{array}\right]=\left[\begin{array}{ccc} N_{j} & c_{1} & <a_{j1,}b_{j1}>\\ & \begin{array}{l} c_{2}\\ \ldots \end{array} & \begin{array}{l} <a_{j2,}b_{j2}>\\ \ldots \end{array}\\ & c_{n} & <a_{jn,}b_{jn}> \end{array}\right]$$

where $N_{j}$ is the evaluation object, $C_{i}$ refers to a certain evaluation feature, and $V_{j}$ is the quantified region of this feature, $C_{i}$ is the set of $c_{i}$, $V_{j}$ is the set of $x_{j}$.

#### 3.2.2. Determination of the Classic Domain Element

The section matter element can be expressed as:

(3) $$\begin{array}{l} R_{P}=\left(P_{0},C_{i},V_{p}\right)=\left[\begin{array}{l} P_{0}\;c_{1}\;\upsilon_{p1}\\ \;\;c_{2}\;\upsilon_{p2}\\ \;\;\cdots \;\cdots \\ \;\;c_{n}\;\upsilon_{\mathrm{pn}} \end{array}\right]=\left[\begin{array}{l} P_{0}\;c_{1}\;<a_{p1},b_{p1}>\\ \;\;c_{2}\;<a_{p2},b_{p2}>\\ \;\;\cdots \;\;\cdots \\ \;\;c_{n}\;<a_{pn},b_{pn}> \end{array}\right] \end{array}$$

Among them, $P_{0}$ is the whole level of the rock mass accident hazards, and $V_{p}$ is the range of all the values of the $C_{i}$ feature, $C_{i}$ is the set of $c_{i}$, $V_{p}$ is the set of $v_{p}$.

#### 3.2.3. Selecting the Object to Be Evaluated

After the corresponding data of the evaluation index is dimensionlessly processed, it is expressed by the matter–element:

(4) $$R=\left[\begin{array}{l} N\;c_{1}\;x_{1}\\ \;\;\;\;c_{2}\;\;\;\;x_{2}\\ \;\;\cdots \;\;\cdots \\ \;\;c_{n}\;\;\;x_{n} \end{array}\right]$$

where *x_n_* is the specific value.

#### 3.2.4. Determine the Relevance of Each Evaluation Indicator

In extension theory, a correlation function is used to indicate the extent to which an element has a certain property. In the following, according to the definition of extension theory, a formula for calculating the correlation degree [30,31,32,33] of each evaluation index is given.

(5) $$k_{j}(x_{i})=\{\begin{array}{ccc} \frac{\rho (x_{i},b,X_{0})}{\rho (x_{i},b,X)-\rho (x_{i},b,X_{0})} & x_{i}\notin X_{0} & \\ \frac{\rho (x_{i},b,x_{0})}{\rho (x_{i},b,X)-\rho (x_{i},b,X_{0})+a_{mji}-b_{mji}} & x_{i}\in X_{0} & \\ \frac{\rho (x_{j},b,X_{0})}{a_{mji}-b_{mji}} & \rho (x_{i},b,X_{0})=\rho (x_{i},b,X) & \end{array}$$

(6) $$\rho (x_{i},b,X_{0})=\{\begin{array}{ccc} a_{mji}-xi & x_{i}<b_{mji} & \\ b_{z} & x_{i}=b_{mji} & \\ x_{i}-b_{mji} & x_{i}>b_{mji} & \end{array}$$

(7) $$\rho (x_{i},b,X)=\{\begin{array}{cc} a_{pi}-x_{i} & x_{i}<b_{pi}\\ \begin{array}{l} b_{z}\\ x_{i}-b_{pi} \end{array} & \begin{array}{l} x_{i}=b_{pi}\\ x_{i}>b_{pi} \end{array} \end{array}$$

#### 3.2.5. Determination of the Evaluation Level

According to the above calculation data and the association degree, the security level is determined. According to the specific value of the association degree, the comprehensive relevance of the three categories of evaluation indicators is calculated, the calculation formula is:

(8) $$k_{j}(U_{m})=\sum_{i=1}^{n}a_{i}k_{j}(x_{i})$$

According to the comprehensive correlation degree, the safety of each type of hazard sources can be pointed out and improvement measures will be proposed.

## 4. Extension Evaluation of E-Zhuang Coal Mine

### 4.1. Overview of E-Zhuang Coal Mine

The E-Zhuang Coal Mine is a subsidiary of Xinwen Mining Group Co., Ltd., located in the southwest of Laiwu Coalfield. The E-Zhuang Mine Industrial Plaza is 1.5 km away from Laiwu City. The geographical coordinates are 117°40′00″ east longitude and 36°11′40″ north latitude. It is located between E-Zhuang Village, Caojiazhuang Village, Shayu Village, and Mawan Village. The mine is about 4 km long from east to west, about 5 km wide from north to south, and has an area of 17.3594 km^2^, the mining depth is 600 m. The coal mining method was the strike long wall retreating coal mining method, and the total caving method was used to manage the roof.

In this evaluation, the 2409 working face was selected. It is located in the west of the second mining area in the West Wing of the mine, where four coal seams are being mined. The elevation of the working face is −520.8 to approximately −480.2 m, the strike length is 370 m, the inclined width is 115 m, and the area is about 40,700 m^2^. The measured working face revealed 16 coal points, all of which can be mined. The coal thickness is 1.55–3.75 m, with an average of 1.60 m. The coal seam dip angle is 18°, and the 2409 transportation chute is arranged along the roof of the four coal seams. The elevation of the roadway is −522.0 to approximately −529.6 m. The roof is supported by bolt, beam, and anchor cable. The two sides are supported by a bolt with a short steel belt and an anchor net. Table 1 shows the characteristics of the roof and floor of the coal seam, and Table 2 shows the sectional characteristics of the roadway.

### 4.2. Calculation of Weights

In this section, the gray correlation analysis method [34,35,36] was used to determine the weight of different indicators. First, the frequency, risk, prevention difficulty, and scope of influence of the above evaluation indicators were obtained, as shown in Table 3.

The correlation coefficient can be calculated according to the following formula:

(9) $$\varepsilon_{i}\left(k\right)=\frac{\min\left|x_{0}^{\prime }\left(k\right)-x_{i}^{\prime }\left(k\right)\right|+\rho *\max\left|x_{0}^{\prime }\left(k\right)-x_{i}^{\prime }\left(k\right)\right|}{\left|x_{0}^{\prime }\left(k\right)-x_{i}^{\prime }\left(k\right)\right|+\rho *\max\left|x_{0}^{\prime }\left(k\right)-x_{i}^{\prime }\left(k\right)\right|}$$

Among them, ρ=0.5, the correlation coefficient is obtained, as shown in Table 4.

Next, the correlation order was calculated. The three types of hazard sources have different effects on the accidents, the Delphi method was used to determine the three coefficients. Therefore, according to their difficulty and degree of danger, based on the accident situation of E-Zhuang coal mine, we made a questionnaire and gave it to ten safety evaluation experts for grading, and finally calculated the coefficient. The coefficients of the three types of hazard sources were 0.8, 0.9, and 0.6.

The correlation order of each hazard source was determined according to the following formula:

(10) $${r^{\prime }}_{0i}=\frac{1}{m}\sum {}_{k=1}^{m}W_{k}\cdot \varepsilon_{i}(k)$$

In the formula, $W_{k}$ is the index of each weight. The calculation formula can be denoted as: Correlation order = (sum of hazard source correlation degree/4) * coefficient. The correlation sequence calculated is the weight coefficient. The results are shown in Table 5.

### 4.3. Extension Safety Evaluation of E-Zhuang Coal Mine

According to the mine safety evaluation system and the actual situation of E-Zhuang coal mine, the classic domain is as shown in Table 6 [37,38]. Meanwhile, the evaluation indicators were scored. We adopted the Delphi method and asked fifteen experts to score the influence value of the indicators. Finally, we summarized and calculated the average value. The scores are shown in Table 7.

The main parameter models used in the evaluation are as follows;

(11) $$R_{P}=(P,C,V)=\left(\begin{array}{ccc} P & U_{11} & \left(0,100\right)\\ & U_{12} & \left(0,100\right)\\ & U_{13} & \left(0,100\right)\\ & U_{14} & \left(0,100\right)\\ & U_{15} & \left(0,100\right)\\ & U_{16} & \left(0,100\right)\\ & U_{17} & \left(0,100\right)\\ & U_{18} & \left(0,100\right)\\ & U_{21} & \left(0,100\right)\\ & U_{22} & \left(0,100\right)\\ & U_{23} & \left(0,100\right)\\ & U_{24} & \left(0,100\right)\\ & U_{25} & \left(0,100\right)\\ & U_{31} & \left(0,100\right)\\ & U_{32} & \left(0,100\right)\\ & U_{33} & \left(0,100\right)\\ & U_{34} & \left(0,100\right)\\ & U_{35} & \left(0,100\right) \end{array}\right)$$

(12) $$R=\left(\begin{array}{ccc} P & U_{11} & 95\\ & U_{12} & 95\\ & U_{13} & 95\\ & U_{14} & 85\\ & U_{15} & 95\\ & U_{16} & 85\\ & U_{17} & 85\\ & U_{18} & 95\\ & U_{21} & 85\\ & U_{22} & 85\\ & U_{23} & 85\\ & U_{24} & 85\\ & U_{25} & 95\\ & U_{31} & 85\\ & U_{32} & 75\\ & U_{33} & 85\\ & U_{34} & 85\\ & U_{35} & 95 \end{array}\right)$$

(13) $$R_{j}=(N,C,V)=\left(\begin{array}{l} \begin{array}{ccccc} & A & B & C & D\\ U_{11} & \left[90,100\right) & \left[80,90\right) & \left[70,80\right) & \left[0,70\right)\\ U_{12} & \left[90,100\right) & \left[80,90\right) & \left[70,80\right) & \left[0,70\right)\\ U_{13} & \left[90,100\right) & \left[80,90\right) & \left[70,80\right) & \left[0,70\right)\\ U_{14} & \left[90,100\right) & \left[80,90\right) & \left[70,80\right) & \left[0,70\right)\\ U_{15} & \left[90,100\right) & \left[80,90\right) & \left[70,80\right) & \left[0,70\right)\\ U_{16} & \left[90,100\right) & \left[80,90\right) & \left[70,80\right) & \left[0,70\right)\\ U_{17} & \left[90,100\right) & \left[80,90\right) & \left[70,80\right) & \left[0,70\right)\\ U_{18} & \left[90,100\right) & \left[80,90\right) & \left[70,80\right) & \left[0,70\right)\\ U_{21} & \left[90,100\right) & \left[80,90\right) & \left[70,80\right) & \left[0,70\right)\\ U_{22} & \left[90,100\right) & \left[80,90\right) & \left[70,80\right) & \left[0,70\right)\\ U_{23} & \left[90,100\right) & \left[80,90\right) & \left[70,80\right) & \left[0,70\right)\\ U_{24} & \left[90,100\right) & \left[80,90\right) & \left[70,80\right) & \left[0,70\right)\\ U_{25} & \left[90,100\right) & \left[80,90\right) & \left[70,80\right) & \left[0,70\right)\\ U_{31} & \left[90,100\right) & \left[80,90\right) & \left[70,80\right) & \left[0,70\right)\\ U_{32} & \left[90,100\right) & \left[80,90\right) & \left[70,80\right) & \left[0,70\right) \end{array}\\ \begin{array}{ccccc} U_{33} & \left[90,100\right) & \left[80,90\right) & \left[70,80\right) & \left[0,70\right)\\ U_{34} & \left[90,100\right) & \left[80,90\right) & \left[70,80\right) & \left[0,70\right)\\ U_{35} & \left[90,100\right) & \left[80,90\right) & \left[70,80\right) & \left[0,70\right) \end{array} \end{array}\right)$$

According to the formula, the correlation degree of each index to the four levels of A, B, C, and D was calculated, as shown in Table 8.

Using formula (8), the environment and equipment factors were calculated and denoted as U_1_ (U_11_, U_12_, U_13_, U_14_, U_15_), the human unsafe behavior was denoted as U_2_ (U_21_, U_22_, U_23_, U_24_, U_25_), the organizational management factor was denoted as U_3_ (U_31_, U_32_, U_33_, U_34_, U_35_). They have four levels of relevance, denoted as levels of A, B, C, D:

(14) $$k_{j}(U_{m})=\max\left\{k_{j}(U_{m})|\left(j=1,2,3,4\right)\right\}\;$$

It can be determined qualitatively that the evaluated object U_m_ belongs to grade J.

(15) $$K\left(U_{m}\right)=\left(\begin{array}{c} K\left(U_{1}\right)\\ K\left(U_{2}\right)\\ K\left(U_{3}\right) \end{array}\right)=\left(\begin{array}{cccc} 0.0817 & -0.0817 & -0.5049 & -0.8596\\ -0.1289 & 0.1289 & -0.2267 & -0.5278\\ -0.1020 & 0.0252 & -0.0905 & -0.2657 \end{array}\right)$$

In order to determine the safety level of the evaluated object more accurately, it is necessary to know the safety degree of the evaluated object inclining to a certain level.

(16) $$\bar{k_{j}}\left(U_{m}\right)=\frac{k_{j}\left(U_{m}\right)-\min k_{j}\left(U_{m}\right)}{\max k_{j}\left(U_{m}\right)-k_{j}\left(U_{m}\right)},$$

(17) $$j^{*}=\frac{\sum_{j=1}^{4}j•\bar{k_{j}}\left(U_{m}\right)}{\sum_{j=1}^{4}\bar{k_{j}}\left(U_{m}\right)},$$

where $j^{*}$ is the characteristic value of the level variable, through it, the degree that the security of the evaluated object inclines to a certain level can be obtained.

The comprehensive weight, *W*_k_, can be calculated from Table 8,

(18) $$W_{k}=\left(\begin{array}{ccc} 0.4656 & 0.3453 & 0.1891 \end{array}\right).$$

The comprehensive correlation degree of the factor U to be evaluated with respect to the four grades of A, B, C, and D is:

(19) $$K_{U}=a\cdot \left[\begin{array}{ccc} K\left(U_{1}\right) & K\left(U_{2}\right) & K\left(U_{3}\right) \end{array}\right]=\left(\begin{array}{cccc} -0.0258 & 0.0112 & -0.3305 & -0.6327 \end{array}\right)$$

In general, the unsafe status of environment and equipment factors (U_1_) belongs to Class A, the unsafe behavior of people (U_2_) belongs to Class B, and the organizational management factor (U_3_) belongs to Class B inclined Class C. The comprehensive safety grade of surrounding rock for the roadway is grade B, as shown in Table 9. This shows that the organization and management of roof accidents really need more attention, and the personnel safety behavior needs to be further improved. Although no serious roof accidents happened in the mine, the scientific management of mine roof accidents should be strengthened. Mine operators should be trained regularly; operators should be strengthened, and regular training should be given to the mine operators. The operators should strengthen their study and enhance their ability to identify the signs of rock accidents in deep roadways. If there are signs of rock accidents, the production should be stopped immediately, and the operators should be evacuated. The support facilities of the mine should be implemented, and the supporting infrastructure should be supported by professional equipment and professional teams, so as to strengthen the monitoring of the roof warning. The potential hazard sources should be checked in time, and some measures should be given to prevent the roof accidents.

## 5. Conclusions

In this paper, three kinds of hazard sources affecting the stability of deep surrounding rock were analyzed, and the evaluation index system of surrounding rock stability was established. According to the extension theory, the evaluation model was put forward and applied in E-Zhuang coal mine. The main conclusions are listed as follows:

(1) The evaluation index system of mine rock accident was established based on the dangerous sources of mine rock accidents. The system includes three kinds of indicators, including the environment and equipment factors, the human factors, and the organizational management factors. Among them, there are fifteen secondary indices, such as support equipment, operator’s age, emergency rescue plan, and safety technical measures. The scientific and comprehensive evaluation index system is helpful to the accuracy of the safety evaluation of mine roadway stability and makes the evaluation results and suggestions more practical.

(2) Because different evaluation indices have different influence on rock mass accidents, it is necessary to process the data and determine their weights. Based on the analysis of the basic situation of E-Zhuang Coal Mine, the weights of the three types of hazard sources of the surrounding rock stability were determined according to the gray correlation analysis method. Combining the theory of hazard sources with the comprehensive evaluation method of extension, the matter–element model of comprehensive evaluation for rock mass accidents in deep roadway was established.

(3) The matter–element model was established by using the extension evaluation method. The safety assessment level was divided into four levels: A (safe), B (generally safe), C (riskier), and D (dangerous). The correlation degree between the assessed matter–element and the four levels was calculated, respectively. The total correlation degree was calculated from three aspects: environment and equipment factors, human factors, and organizational management factors, so as to determine the safety level of the whole system. The assessment model was applied to the E-Zhuang coal mine, and finally the following conclusions were obtained: the evaluation level of environment and equipment was "A", which means safe. The evaluation level of human factor is "B", which means generally safe. The evaluation level of organizational management factor is "B", which means generally safe.

## Figures and Tables

**Table 1:** Characteristic of coal mine floor and roof.

Name	Rock	Thickness/m	Rock Characteristics
Main roof	Medium grained sandstone	20	Gray–white; its main ingredient is quartz, followed by feldspar; calcareous cementation, dense and hard, not easy to fall
Immediate roof	Fine sandstone and siltstone	5	Light gray; its main ingredient is quartz, followed by feldspar; calcareous cementation, relatively hard and easy to fall; the compressive strengths Rc = 62.0 MPa; joint crack spacing I = 0.5 m; layered thickness h = 0.2 m
False roof	Carbonaceous mudstone	0–0.5	Lamellar; firmness coefficient f = 3
Coal seam	4# coal	1.6	Relatively stable thickness
Immediate floor	Mudstone	2.0	Light gray; silty sand; no bedding; relatively hard; not easy to deform; allowable pressure ratio 26.5 MPa, the compressive strengths Rc = 39.27 MPa

**Table 2:** Roadway section characteristics.

Name of Roadway	Roadway Shape	Height of Roadway/m	Roadway Section Area/m^2^
2409 working face transportation trough	trapezium	2.25	7.5

**Table 3:** Data on three major types of hazard sources.

Source of Risk	Frequency	Risk	Prevention Difficulty	Sphere of Influence
Compression strength of surrounding rock	0	0	2	1
Rock quality designation (RQD)	1	0	0	1
Support equipment	3	0	4	0
Roof conditions	3	1	4	2
Hydrogeological conditions	1	0	1	0
The roadway’s size and shape	2	1	2	0
The depth of excavation	3	1	2	1
Exploitation influence of workings nearby	2	0	1	0
Staff safety awareness	0	0	1	0
length of service	1	0	2	0
Personal protection	3	0	2	0
Emergency self-rescue ability	4	0	4	0
Staff certification	4	0	4	1
Related rules and regulations	3	1	2	2
Safety education and training	3	2	4	0
Security management organization	1	0	1	0
Emergency rescue plan	2	1	3	1
Safety technical measures	2	1	2	1

**Table 4:** Correlation coefficient table.

Source of Risk	Frequency of Occurrence	Risk	Prevention Difficulty	Sphere of Influence
Compression strength of surrounding rock	1.000	1.000	0.500	0.667
Rock quality designation (RQD)	0.667	1.000	1.000	0.667
Support equipment	0.400	1.000	0.333	1.000
Roof conditions	0.400	0.667	0.333	0.500
Hydrogeological conditions	0.667	1.000	0.667	1.000
The roadway’s size and shape	0.500	0.667	0.500	1.000
The depth of excavation	0.400	0.667	0.500	0.667
Exploitation influence of workings nearby	0.500	1.000	0.667	1.000
Staff safety awareness	1.000	1.000	0.667	1.000
length of service	0.667	1.000	0.500	1.000
Personal protection	0.400	1.000	0.500	1.000
Emergency self-rescue ability	0.333	1.000	0.333	1.000
Staff certification	0.333	1.000	0.333	0.667
Related rules and regulations	0.400	0.667	0.500	0.500
Safety education and training	0.400	0.500	0.333	1.000
Security management organization	0.667	1.000	0.667	1.000
Emergency rescue plan	0.500	0.667	0.400	0.667
Safety technical measures	0.500	0.667	0.500	0.667

**Table 5:** Correlation sequence calculation results.

Source of Risk	Correlation Sequence (a)
Compression strength of surrounding rock	0.6334
Rock quality designation (RQD)	0.6668
Support equipment	0.5466
Roof conditions	0.3800
Hydrogeological conditions	0.6668
The roadway’s size and shape	0.5334
The depth of excavation	0.4468
Exploitation influence of workings nearby	0.6334
Staff safety awareness	0.8251
length of service	0.7126
Personal protection	0.6525
Emergency self-rescue ability	0.5999
Staff certification	0.5249
Related rules and regulations	0.3101
Safety education and training	0.3350
Security management organization	0.5001
Emergency rescue plan	0.3351
Safety technical measures	0.3501

**Table 6:** Classical domain table of mine rock accidents.

Evaluation Feature	Grading standards			
	Safe (A)	Generally Safe (B)	Riskier (C)	Dangerous (D)
Compression strength of surrounding rock	[90, 100)	[80, 90)	[70, 80)	[0, 70)
Rock quality designation (RQD)	[90, 100)	[80, 90)	[70, 80)	[0, 70)
Support equipment	[90, 100)	[80, 90)	[70, 80)	[0, 70)
Roof conditions	[90, 100)	[80, 90)	[70, 80)	[0, 70)
Hydrogeological conditions	[90, 100)	[80, 90)	[70, 80)	[0, 70)
The roadway’s size and shape	[90, 100)	[80, 90)	[70, 80)	[0, 70)
The depth of excavation	[90, 100)	[80, 90)	[70, 80)	[0, 70)
Exploitation influence of workings nearby	[90, 100)	[80, 90)	[70, 80)	[0, 70)
Staff safety awareness	[90, 100)	[80, 90)	[70, 80)	[0, 70)
length of service	[90, 100)	[80, 90)	[70, 80)	[0, 70)
Personal protection	[90, 100)	[80, 90)	[70, 80)	[0, 70)
Emergency self-rescue ability	[90, 100)	[80, 90)	[70, 80)	[0, 70)
Staff certification	[90, 100)	[80, 90)	[70, 80)	[0, 70)
Related rules and regulations	[90, 100)	[80, 90)	[70, 80)	[0, 70)
Safety education and training	[90, 100)	[80, 90)	[70, 80)	[0, 70)
Security management organization	[90, 100)	[80, 90)	[70, 80)	[0, 70)
Emergency rescue plan	[90, 100)	[80, 90)	[70, 80)	[0, 70)
Safety technical measures	[90, 100)	[80, 90)	[70, 80)	[0, 70)

**Table 7:** Evaluation index score.

Evaluation Feature	Score
Compression strength of surrounding rock	95
Rock quality designation (RQD)	95
Support equipment	95
Roof conditions	85
Hydrogeological conditions	95
The roadway’s size and shape	85
The depth of excavation	85
Exploitation influence of workings nearby	95
Staff safety awareness	85
length of service	85
Personal protection	85
Emergency self-rescue ability	85
Staff certification	95
Related rules and regulations	85
Safety education and training	75
Security management organization	85
Emergency rescue plan	85
Safety technical measures	95

**Table 8:** Correlation degree calculation results.

Evaluation Project	K(A)	K(B)	K(C)	K(D)	Weight Coefficient
U11	0.0500	−0.0500	−0.1364	−0.2083	0.6334
U12	0.0500	−0.0500	−0.1364	−0.2083	0.6668
U13	0.0500	-0.0500	−0.1364	−0.2083	0.5466
U14	−0.0556	0.0556	−0.0556	−0.1500	0.3800
U15	0.0500	−0.0500	−0.1364	−0.2083	0.6668
U16	−0.0556	0.0556	−0.0556	−0.1500	0.5334
U17	−0.0556	0.0556	−0.0556	−0.1500	0.4468
U18	0.0500	−0.0500	−0.1364	−0.2083	0.6334
U21	−0.0556	0.0556	−0.0556	−0.1500	0.8251
U22	−0.0556	0.0556	−0.0556	−0.1500	0.7126
U23	−0.0556	0.0556	−0.0556	−0.1500	0.6525
U24	−0.0556	0.0556	−0.0556	−0.1500	0.5999
U25	0.0500	−0.0500	−0.1364	−0.2083	0.5249
U31	−0.0556	0.0556	−0.0556	−0.1500	0.3101
U32	−0.1667	−0.0625	0.0625	−0.0625	0.3350
U33	−0.0556	0.0556	−0.0556	−0.1500	0.5001
U34	−0.0556	0.0556	−0.0556	−0.1500	0.3351
U35	0.0500	−0.0500	−0.1364	−0.2083	0.3501

**Table 9:** Comprehensive correlative degree.

K_A_(U)	K_B_(U)	K_C_(U)	K_D_(U)	Grade
−0.0258	0.0112	−0.3305	−0.6327	B
